# A Taguchi Approach for Optimization of Antimicrobial Effect of Whey Protein Based Edible Film Fermented by *Bacillus clausii*

**DOI:** 10.3390/polym16233375

**Published:** 2024-11-29

**Authors:** Ali Raza Khan, Elif Sezer, Özge Aslan, Arzu Çağrı-Mehmetoglu

**Affiliations:** Department of Food Engineering, Faculty of Engineering, Sakarya University, Sakarya 54187, Turkey; jordanjaxx4213@gmail.com (A.R.K.); elifsezer@sakarya.edu.tr (E.S.); oz_gem_03@hotmail.com (Ö.A.)

**Keywords:** antimicrobial edible film, biocontrol, antagonistic bacteria, *Bacillus clausii*

## Abstract

*Bacillus clausii*, an antagonistic bacterium, was utilized to develop antimicrobial edible films based on whey protein concentrate. This study employed a Taguchi test (3 × 3) to evaluate the impact of temperature, pH, and protein concentration on film properties. Optimal growth of *B. clausii* occurred at 6% (*w*/*v*) protein and pH 9.5. The resulting film solutions demonstrated antimicrobial activity, exhibiting inhibition zones against *Aspergillus niger*, *Penicillium expansum*, *Staphylococcus aureus*, and *Escherichia coli*, with inhibition zone diameters of 13.68 mm, 16.88 mm, 11.38 mm, and 17.15 mm, respectively. The optimum antimicrobial property of the films was observed when the incubation condition of pH 8.5, 35 °C and 6% (*w*/*v*) protein. Survival rates of *B. clausii* in the dry film were 86% at 4 °C and 87% at 25 °C over 14 days. Additionally, the highest tensile strength (TS) and percent elongation at break (%E) for the films were recorded at 3.14 MPa (pH = 9.5, 37 °C, 8% protein) and 27.63% (pH = 9.0, 35 °C, 10% protein), respectively. These findings demonstrate the potential for developing effective antimicrobial films through 24-h fermentation of *B. clausii* in the film solution. This antimicrobial film shows potential for use in wound dressings or food packaging applications.

## 1. Introduction

Food products’ safety and quality are determined by the number and type of spoilage and pathogenic bacteria existing on the food surface [1]. Antimicrobial edible packaging is one of the preservation methods that can extend the product’s shelf-life while ensuring the safety of consumers [2]. The popularity of edible films and coatings based on whey protein concentrate (WPC) has grown along with environmental awareness [3]. The production of films based on WPC has been studied specifically because of their numerous advantageous properties, including high elasticity, good appearance, and moderate oxygen barrier. Furthermore, these films perform several tasks besides being edible packaging material, such as acting as a carrier for nutraceuticals, antimicrobials, or antioxidants [4]. In recent years, with the increasing awareness of consumers of natural antimicrobials, studies in this field have started to attract attention in the antimicrobial film research area [5]. Antagonistic microorganisms are among the natural antimicrobials that are also used as bio-control agents.

Antagonism microorganisms frequently change their surroundings along with their metabolic products, making it difficult for other competing organisms to survive [6]. Microbial antagonists, which are environmentally friendly and resistant to harsh conditions, can be used to protect produce against pre-harvest diseases and against microbial spoilage in storage.

The *Bacillus* genus of bacteria generates a variety of antimicrobial compounds, such as *B. clausii* strains, producing protease and bacteriocins with action against Gram-positive bacteria. *B. clausii* can be considered for use as an antagonistic agent since it has so many characteristics as a spore-producing bacteria: rapid growth in liquid culture, extreme thermal tolerance, and ability to form a resistance [7,8].

While whey protein concentrate (WPC) films have gained popularity as edible packaging materials due to their biodegradability and beneficial mechanical properties, there is a limited understanding of how to optimize the incorporation of natural antimicrobial agents, particularly those produced by *B. clausii*, within these films. Current research on WPC-based antimicrobial films largely focuses on synthetic or plant-based antimicrobial compounds, with minimal attention given to microbial antagonists, such as *B. clausii*, that offer an eco-friendly and sustainable alternative. Furthermore, little is known about the effects of varying pH, temperature, and protein content in the WPC film solution on the production and efficacy of antimicrobial compounds generated by *B. clausii*. These factors are crucial, as they influence both the antimicrobial activity and the physical properties of the resulting film, including water vapor permeability and mechanical strength, which are key for practical applications in food packaging and wound dressings. This study addresses this gap by systematically investigating the role of environmental conditions in optimizing *B. clausii*-based antimicrobial production and integrating it effectively into WPC films, thereby enhancing their potential for safe, natural, and sustainable food preservation.

The aim of the study was to investigate the effect of pH, temperature, and protein content on antimicrobial production by *B. clausii* in the WPC film solution and on the properties (mechanical water vapor permeability and antimicrobial properties) of the resultant WPC film.

## 2. Materials and Methodology

### 2.1. Materials

NaOH, HCl, glycerin, ammonium sulfate, silica gel balls, and Whatman no:1 filter paper were supplied from a Sigma-Aldrich (Burlington, VT, USA). The whey protein concentrate (80%) was provided by Malkara Birlik Milk and Dairy Products (Sakarya, Turkey). Tryptic Soy Agar (TSA), Tryptic Soy Broth (TSB), Casein Pepton Agar, and Mueller Hilton Agar were supplied by Merck (New York, NY, USA). The microbial strains *B. clausii* (O/C, T, SIN, and N/R), *S. aureus* ATCC 25923, *P. expansum* MRC 200806, *A. niger* MRC 200806, and *E. coli* O157:H7 NCTC 12900 were supplied from the culture collection of the Department of Food Engineering at Sakarya University (Serdivan, Turkey).

### 2.2. Applications of the Taguchi Trial Model

The Taguchi trial model (L9, 3^3) was designed to investigate the optimum production of antimicrobials by *B. clausii* and the optimum properties of the films regarding 3 different factors (pH, temperature, and the amount of protein in the film solution) at 3 effective levels (Table 1).

### 2.3. Culture Preparation

The *B. clausii* strain used in this study was grown in TSB at 30 °C for 24 h. The bacterial cell was collected using a centrifuge (Hettich Universal 320R, Tuttlingen, Germany) at 9000 rpm for 15 min at 4 °C. The following supernatant was removed and the bacterial cells were washed two times with sterile distilled water.

### 2.4. Preparation of Films

6, 8, or 10 g of WPC was dissolved in 91, 88, or 85 mL distilled water on a magnetic stirrer, respectively, then the pH was adjusted to 8.5, 9.0, and 9.5 with 0.1 M NaOH and HCl [9]. Further, the solution was put in the water bath for 30 min at 90 °C, adding 3, 4, and 5 mL glycerol to the solution with 6, 8, or 10 g WPC in the last 5 min, respectively. After cooling, 1 mL of *B. clausii* culture (7.2 ± 0.2 log_10_ CFU/mL vegetative cells, 6.9 log ± 0.09 log_10_ CFU/mL spore cells) in the pellet was transferred to each WPC film solution. Then, the solutions were incubated at 33 °C, 35 °C, and 37 °C for 24 h. After 24 h, 100 mL of solution was cast onto the Teflon-coated plates (diameter of 22 cm^2^) and air bubbles were removed through the hot loop. Following drying under a biological safety cabinet at 50 ± 5% RH at 25 °C ± 1 for 48 h, they were peeled from the plates.

### 2.5. Mechanical Properties of the Films

The thickness of the films was evaluated to the closest 0.001 mm using a micrometer on the five different points (Pittsburgh, PA, USA). Using Instron Universal Testing 3367 (Ames, IA, USA) and the Bluehill 2®2 software, tensile strength (TS) and percent elongation at break (% E) were measured using the standard method ISO 1924-2-1994 (Norwood, MA, USA) [10].

### 2.6. Water Vapor Permeability of the Films

The water vapor permeability (WVP) of the films was measured using the standard American Society for Testing and Materials (ASTM 96-80) [11] method with a few changes [9]. The film in diameter of 45 cm was sealed on the mouth of the small cups (40 mm in diameter, 25 mm in depth) containing 10 g silica gel balls, and then the cups were placed in a conditioning chamber at 85% RH, at 37 °C. The weight of the cups was observed every 2 h up to 12 h. WVP of the films was calculated using Equation (1).

WVP = (C × T) × (A × Δp)^−1^ g∙mm∙m^−2^∙d^−1^kPa^−1^ (1)

In the equation, A is the exposed film area (0.00126 m^2^), T is the film thickness (mm), p is the vapor partial pressure difference across the film (p: kPa at 85% RH at 37 °C), and the water vapor transmission rate (g/d).

### 2.7. Water Solubility of the Films

By the procedures outlined in Karabulut and Cagri-Mehmetoglu [9], the film’s percent water solubility (% WS) was determined. After being cut into 2 × 2 cm^2^ squares, the film samples underwent a second drying process at 105 ± 2 °C for 24 h. Before and following drying, each sample’s weight was calculated. The pieces were then submerged in 50 mL of distilled water for 24 h at 23 ± 2 °C, using filter paper (Whatman, Grade 1, Sigma-Aldrich) to filter the solution; the film’s water-insoluble component was obtained. After being weighed and dried at 105 ± 2 °C for 24 h, the paper with the water-soluble component was again weighed. Utilizing Equation (2), the % WS was determined.

% WS = (m_1_ − m_2_) × (m_1_)^−1^ × 100 (2)

In Equation (2), m_1_ and m_2_ are the initial and final dried weights of the film samples, respectively.

### 2.8. Microbiological Analysis of the Film Solutions and the Dry Film Samples

After fermentation, the edible film solution (1 mL) was diluted in 9 mL NaCl solution (0.85%) and dry film samples (0.1 g) were homogenized in 9.9 mL NaCl solution (0.85%) using a stomacher for 2 min. After serial dilution using 9 mL of NaCl solution (0.85%), 0.1 mL of culture was plated on TSA and incubated at 30 °C for 24 h to investigate the total vegetative cell count of *B*. *clausii*. To observe spore count in the prepared film solutions or dry film samples, the serial dilutions were heated in a water bath at 80 °C for 5 min, cooled immediately, and plated on Casein Pepton Agar. Following incubation at 30 °C for 24 h, the colonies on the plate were counted. The number of vegetative and spore cells of *B. clausii* in the dry film was also determined using the same method after 14 days of storage at 4 °C and 25 °C.

### 2.9. Antimicrobial Properties of the Film Solution

The film solution, which was left to ferment for 24 h with *B. clausii* (7.2 log CFU/mL) at 33, 35, or 37 °C, was centrifuged at 9000 RPM at 4 °C for 15 min, then the supernatant (10 mL) was saturated with ammonium sulfate (6 g) and kept at 4 °C for 24 h. The solutions were then centrifuged at 9000 RPM at 4 °C for 15 min and washed twice with distilled water, after which the pellet was dissolved in 1 mL of 100 mM Tris-HCl buffer (pH 7.0).

The disk diffusion method determined the antimicrobial activity of the precipitated proteins [12]. In this method, 10 µL of the precipitated proteins were sucked by sterilized filter paper disks (6 mm in diameter) and placed on the surface of Mueller Hilton Agar inoculated by *E. coli*, *S. aureus*, *P. expansum*, and *A. niger.* The plates were incubated at 35 °C for 24 h for bacteria cultures and 25 °C for 5 days for mold cultures. Inhibition zones around discs were measured perpendicularly using a ruler.

### 2.10. Statistical Analysis

Three replicates were used for statistical analysis using the SPSS program version 26.0 (IBM). The expression of values such as mean standard deviation (SD) and evaluation of mean was carried out using variance (ANOVA).

## 3. Results and Discussion

### 3.1. The Growth of B. clausii in the Film Solution

**Figure 1:**
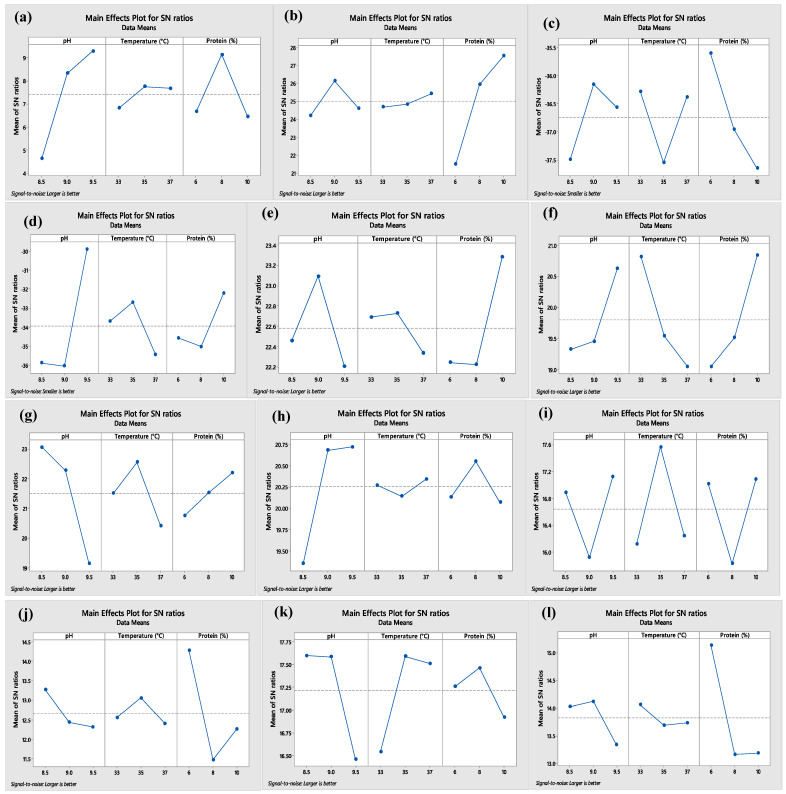
Signal/noise ratio results of tensile strength (**a**), percent elongation (**b**), water vapor permeability (**c**), percent water solubility of the film containing *B. clausii* (**d**), inhibition of the film containing *B. clausii* against *A. niger* (**e**), *P. expansum* (**f**), *E. coli* (**g**), *S. aureus* (**h**), the growth of *B. clausii* vegetative cells in the film solution (**i**), the growth of *B. clausii* spore cells in the film solution (**j**), the number of *B. clausii* vegetative cells in the dry film (**k**), the number of *B. clausii* spore cells in the dry film (**l**) in the Taguchi experimental design.

After 24 h fermentation, the highest number of vegetative and spore forms of *B. clausii* in the WPC edible film solution was counted at 8.96 and 6.07 log CFU/mL, respectively. The optimum growth of *B. clausii* (total of vegetative and spore cells, 7.92 log CFU/mL) was observed when the pH of the film solution was 9.5, the incubation temperature was 35 °C, and the protein content in the film was 6% (Table 2). Incubation temperature was the most effective parameter for the growth of *B. clausii* in the film solution (Figure 1i). In previous studies, unlike our study, optimum growth temperature and pH value for *B. clausii* were reported as 37 °C and pH 8 [13]. The nutrition value of the film solution might shift its growth requirement. The studies have also shown that *B. clausii* has an alkalophilic character; this must be why it showed better growth at pH 9.5 in this study compared to low pH conditions [14].

### 3.2. The Survival of B. clausii in the Films

After drying the films, vegetative and spore cells were reported as 8.18 and 7.44 log CFU/g, respectively. The best survival and growth ratio of vegetative and spore cells of *B. clausii* in the dry film at 25 °C for 14 days was observed as 87 and 100%, respectively (Table 3). The survival of *B. clausii* in vegetative form over 14 days was supported as similar at both storage temperatures. The highest number of vegetative *B. clausii* cells was reported when the pH of the film was 9.5 and protein content was 6% (Supplementary Figure S1a,b). According to the Taguchi test trial, pH and protein content in the film formula were the most influential parameters regarding the survival of *B. clausii* vegetative cells at 4 and 25 °C, respectively. On the other hand, changes in pH most effectively change the survival ability of the spores at both temperatures (Supplementary Figure S1c,d). As mentioned in Section 3.1, studies have also demonstrated the alkalophilic nature of *B. clausii*, which is likely to have explained its improved survival at pH 9.5 observed in this study, compared to its performance under low pH conditions [14].

Available water content in the film is an important factor for the survival and growth of microorganisms. When the protein content in the film was 6% compared with 10%, better survival ability for *B. clausii* was observed. The most important reason for this might be that the amount of free water decreases indirectly when the amount of whey protein increases. Whey protein as a water-soluble protein has a high binding affinity towards water molecules. Increasing the amount of protein in the film formula could directly affect the survival ratio of *B. clausii*. Bacteria cells cannot grow in the environment with water activity lower than 0.76 and the water activity of the WPC film is usually changing from 0.61 to 0.64 [9]. However, some deep sea halophilic eubacteria, like *B. clausii,* can resist in a dry film environment by producing compatible solutes, like ectoine [15]. At a high osmatic pressure, *B. clausii* is able to maintain its internal osmotic pressure using ectoine. In addition, the survival ratio of *B. clausii* in the WPC film was similar to that of *B. coagulans* in the milk protein-based film (87%) in similar conditions [16].

Research indicates that the survival ability of *B. clausii* spores is not significantly influenced by storage temperature, due to their spore-forming nature, which provides robust stability across various conditions. This stability is supported by studies that emphasize the spores’ resistance to environmental stress, including room-temperature storage [17,18]. For example, *B. clausii* spores showed consistent viability in experiments designed to test their stability under different storage and gastrointestinal conditions. These studies reinforce the strain’s effectiveness as a probiotic under diverse handling and storage scenarios [18,19].

### 3.3. Mechanical Properties of Films

The thickness of WPC film containing *B. clausii* did not change significantly according to parameters such as temperature, pH, and the protein concentration of the film with *p* > 0.05 (Table 4). Protein content significantly influences TS, especially when combined with pH and temperature changes (*p* < 0.05). Optimum TS (3.14 MPa) was observed at 8% WPC at pH 9.5 and 37 °C. %E is influenced primarily by pH and temperature rather than protein concentration alone, as the combination effect is significant (*p* < 0.05). Maximum %E (%27) was achieved with 10% WPC at pH 9, 35 °C. According to the Taguchi program, TS and %E were significantly affected by pH and protein content, respectively (*p* < 0.05) (Figure 1a,b) (Supplementary Table S1). Combination effect of pH and temperature, protein concentration and temperature, and all three parameters on TS were also found to be significant (*p* < 0.05). However, only combination of pH and temperature significantly affected %E of the film (*p* < 0.05).

In our previous study, when *Williopsis saturnus* yeast was added to WPC-based film, the TS and %E were measured as 5.6 MPa and 23%, respectively [9]. An increase in yeast numbers in the film did not significantly affect the TS, but decreased percent elongation was observed. The TS of the film measured in the current study was reported to be smaller and the E% was higher than in the previous study. The reason for the decrease in the film’s strength in this study is thought to be that the protease enzyme synthesized during the 24-h fermentation of *B. clausii* in the film solution degraded part of the protein content. Thus, shortening the length of the protein chains that make up the structure of the film would also allow also strength reduction and elasticity improvement. The presence of bacteria might also support % E and reduce the strength, probably by acting as a plasticizer [9].

The present study observed the optimum TS at the highest pH (pH 9.5) condition. In the production of whey protein film, denaturation is carried out by breaking the hydrogen bonds in the protein structure by heating. This process also allows the release of hydrophobic and internal SH groups. The film is formed by forming new hydrogen and hydrophobic bonds, and disulfide bonds between protein chains during drying [20]. Studies have shown that disulfide bond formations between protein chains increase above pH 8 [21]. Therefore, increased disulfide bonds in the film at pH 9.5 have improved the film’s strength.

One of the limitations in this study is that the tensile strength and percent elongation at break may be insufficient for certain applications, where mechanical durability is essential, particularly in food packaging. Food packaging requires materials with high tensile strength and sufficient elongation at break to withstand mechanical stress during production, transportation, and handling. The tensile strength of recently studied antimicrobial edible films based on various materials, such as gelatin, chitosan, and starch, has been reported to range from 5 to 60 MPa [22,23,24]. Studies have also shown that the mechanical properties of WPC-based films can be significantly enhanced by adding reinforcing fillers or crosslinkers, such as nano-clays, nanoparticles, or genipin [22,25]. For instance, Al-Hayalı and Cagri-Mehmetoglu [22] demonstrated that incorporating iron nanoparticles into WPC films increased their tensile strength from 2.42 to 5.82 MPa. In future studies, these recommended modifications are expected to further improve mechanical strength and mitigate current limitations.

These results demonstrate the importance of optimizing processing parameters to achieve the desired mechanical properties in WPC films containing *B. clausii*. The lack of significant variation in thickness across conditions indicates that such films could provide consistent performance in applications requiring durability, such as food packaging or probiotic delivery systems.

### 3.4. Water Vapor Permeability (WVP) of Films

The optimum WVP of the film sample at 45.89 g·mm·m^−2^·d^−1^kPa^−1^ was reported at the condition with pH 9, 37 °C and 6% WPC (Table 4). According to the Taguchi program, protein contents in the film formula had the highest effect on WVP of the film (Figure 1c). WVP was significantly improved by decreasing the amount of protein in the film (*p* < 0.05). Moreover, pH and temperature were also effective parameters on WVP. Interaction effects of all parameters on WVP were also found to be significant (*p* < 0.05) (Supplementary Table S1). As mentioned above, high pH promotes the formation of disulfide bonds and reduces the gap between chains in protein films. Thus, it increases the film’s strength and prevents the permeation of water vapor. Muñoz et al. [26] also reported that increasing pH from 7 to 10 in the film formulation based on WPC and chia mucilage improved its WVP.

Compared to similar studies of WPC-based film, the WVP of the film was found to be approximately two times higher in this study [9]. In addition, due to the fact that the films made with WPC have high water vapor permeability due to its hydrophilic feature, the whey proteins broken down by the protease enzyme produced by *B. clausii* in this study may weaken the interaction between the protein chains and further reduce the water vapor barrier of the film.

The film’s WVP is relatively high, which could reduce its effectiveness as a moisture barrier. Potential approaches to mitigate high WVP could include incorporating low levels of hydrophobic compounds (such as waxes, essential oils, or nano-clays), which have been shown to lower WVP while maintaining other film properties [27]. Another recommendation for future studies is to explore multi-layered film designs or coatings, as these methods are often effective in enhancing barrier properties in moisture-sensitive applications [28].

### 3.5. Percent Water Solubility of Films

Percent water solubility (%WS) of the WPC film containing *B. clausii* ranged from 24.49 to 80.02%, with the lowest %WS measured when the WPC in the film was 10% (wt/v), incubation temperature was 35 °C and the pH of the film before fermentation was 9.5 (Table 4, Figure 1d). The pH or protein increase in the film formula significantly improved the %WS of the films (*p* < 0.05); however, higher or lower than 35 °C incubation temperature decreased %WS properties. The pH content of the film was the most significant parameter for the %WS of the film. Effect of temperature and protein content together on %WS was also significantly important (*p* < 0.05) (Supplementary Table S1). Compared to other similar studies, the water solubility of the films produced in this study was relatively higher [9]. One of the reasons might be that the studies showed that incorporating bacteria or yeast into protein film formula could significantly increase solubility, since adding the cells weakens the interaction between intermolecular protein chains. For example, Karabulut and Cagri-Mehmetoglu [9] reported that incorporating *W. saturnus* yeast cells into WPC-based film significantly increased the solubility of the film from 23 to 36%. The other reason may be that protein hydrolysis by bacterial enzymes produced by *B. clausii* during 24 h fermentation increased the amount of low molecular weight proteins and peptides in the film texture. This will increase the number of film-to-water transitions, along with solubility.

### 3.6. Antimicrobial Effect of the Film Solution

The film solution based on WPC containing *B. clausii* showed the inhibition effects on the molds (*A. niger*, and *P. expansum*) and the bacteria (*S. aureus*, and *E. coli O157:H7*). The highest diameter of the inhibition zones against the growth of *A. niger* and *P. expansum* was reported as 13.63 and 16.88 mm, respectively (Table 5). The optimum inhibition against mold was observed when the incubation temperature was 35 °C, the pH of the film solution was 8.5, and protein content in the film formula was 8% (*w*/*v*). The pH was the most effective parameter for the antifungal properties of the film solution (Figure 1e,f). While the diameter of inhibition zones against the growth of *E. coli* O157:H7 ranged from 7.38 to 17.25 mm, the optimum antimicrobial properties were received against *E. coli* when the incubation temperature was 33 °C and protein content was 10% (Figure 1g). Furthermore, the growth of Gram-positive *S. aureus* was inhibited by WPC film solution, with inhibition zones changing in the range of 8.88 mm to 11.38 mm. According to the Taguchi test trial, pH was the most effective parameter for the antimicrobial properties of the film solution against *S. aureus* (Figure 1h).

In this study, *B. clausii* was allowed to grow and produce bacteriocins in the film solution for 24 h. The studies have reported that clausin, one of these bacteriocins produced by *B. clausii*, inhibits peptidoglycan synthesis, and inhibits Gram-positive bacteria [29]. Whey proteins are an important nutritional source containing essential amino acids [30]. As shown in the previous studies, *B. clausii* antimicrobial agent production was achieved by using whey as a medium [31]. Other than bacteriocin *B. clausii* synthesis, some antimicrobial peptides might also be produced when hydrolyzing whey protein by protease enzymes during fermentation. These antimicrobial hydrolyzed peptides also have antibacterial effects against Gram-positive and Gram-negative bacteria, including *Salmonella* Typhimurium, *E. coli*, *Shigella flexneri*, *S. aureus*, *Listeria monocytogenes*, and *Enterococcus faecalis.*

The findings of this study underscore the potential of using *B. clausii* in the development of antimicrobial films, particularly in the food packaging industry. The observed inhibition of both molds and pathogenic bacteria suggests that these films could play a significant role in enhancing food safety by reducing spoilage and preventing foodborne illnesses. The ability of the film to maintain its antimicrobial properties at different pH levels and temperatures also indicates its versatility for various food products.

## 4. Conclusions

This study successfully demonstrates the multifaceted applications of *B. clausii*-enriched WPC films, highlighting their potential in food packaging and probiotic delivery. Optimal growth of *B. clausii* (vegetative and spore forms) occurred at pH 9.5, 35 °C, and 6% protein concentration. The film’s nutrient content appeared to modify the microorganism’s growth conditions, promoting its alkalophilic behavior.

*B. clausii* was able to grow in the film based on WPC at all tested conditions and produce antimicrobial substances. The antimicrobial produced in the fermented solution of the film was able to inhibit the growth of *A. niger*, *P. expansum*, *E. coli*, and *S. aureus*. The findings suggest that these antimicrobial films can effectively inhibit the growth of common food spoilage microorganisms, as well as pathogens. Such capabilities indicate their potential application in the food packaging industry, enhancing food safety by prolonging shelf life and reducing the risk of foodborne illnesses. This study showed that it is possible to produce a film solution containing antimicrobial substances by fermenting *B. clausii*. WPC-based edible films could have the ability to provide surface protection of food products when they contain antimicrobial substances. They could enhance food safety by prolonging shelf life and reducing the risk of foodborne illnesses. The survival of vegetative cells (~87%) and spores (100%) was unaffected by storage temperature, underscoring the stability of *B. clausii* spores under diverse conditions. This resilience aligns with findings on spore-forming bacteria’s resistance to environmental stressors.

While film thickness remained consistent across various conditions, TS and elongation at %E were optimized at pH 9.5, 37 °C, and 8% WPC for TS (3.14 MPa), and at pH 9, 35 °C, and 10% WPC for %E (27%). The presence of *B. clausii* and protease activity is likely to have acted as a plasticizer, reducing film strength but improving elasticity. Limitations in TS and %E suggest the need for additional reinforcement strategies, such as adding nanofillers or crosslinkers. Water vapor permeability (WVP) was influenced by protein content, pH, and temperature. The high WVP, attributed to the hydrophilic nature of WPC and enzymatic activity, can be mitigated by incorporating hydrophobic additives or multilayer designs for enhanced moisture resistance. Films also exhibited relatively high water solubility (%WS), influenced by pH, protein content, and bacterial activity. The increased solubility was likely to be due to hydrolyzed proteins and weakened intermolecular interactions, consistent with previous studies on microorganism-enriched films.

This study demonstrated an economical alternative by producing antimicrobials directly in the film solution through *B. clausii* fermentation, rather than adding external antimicrobials. These active WPC films, containing live antagonistic and probiotic bacteria, can be used to extend the shelf life of commercially sliced products, like cheese or deli meats, and to protect against contamination.

Furthermore, the antimicrobial properties of these films could be valuable in developing wound dressings to prevent infections, support faster healing, and reduce antibiotic use. The films’ composition, based on whey protein (GRAS) and *B. clausii* probiotics, also opens possibilities for oral medical applications, such as dissolvable antimicrobial strips or probiotic delivery systems for the gut.

However, this study has several potential limitations. First, the applicability of the results to industrial-scale production may be questionable, as different outcomes could arise in large-scale manufacturing processes. Another concern is that the use of antimicrobial films containing bacteria could negatively affect the taste, smell, or appearance of food products, potentially influencing consumer acceptance. Addressing these weaknesses is essential for guiding future research and enhancing product development. Moreover, simulations of actual food storage could be used to evaluate the films’ performance in real-world situations.

## Figures and Tables

**Table 1:** Design of Taguchi trial model (L9, 3^3).

Sample Code	pH of the Film Solution BI (AI) *	Incubation Temperature (°C)	Whey Protein in the Film Solution (%) (*w*/*v*)
1	8.5 (7.52 ± 0.5)	33	6
2	8.5 (7.63 ± 0.9)	35	8
3	8.5 (6.94 ± 0.7)	37	10
4	9.0 (8.09 ± 0.9)	33	8
5	9.0 (7.96 ± 1.1)	35	10
6	9.0 (8.00 ± 0.6)	37	6
7	9.5 (8.16 ± 1.2)	33	10
8	9.5 (8.43 ± 0.8)	35	6
9	9.5 (8.65 ± 0.5)	37	8

* BI: before incubation, AI: after 24 h of incubation.

**Table 2:** The number of vegetative and spore cells of *B. clausii* (log_10_ CFU/g) during WPC film preparation and 14 days of storage.

Vegetative Cells			Dry Films (log_10_ CFU/g)		
				4 °C	25 °C
Sample Code **	BI * (log_10_ CFU/mL)	AI * (log_10_ CFU/mL)	0 Day	14 Day	
1	7.2 ± 0.13 ^a^	7.81 ± 0.12 ^d^	8.18 ± 0.18 ^e^	7.07 ± 0.44 ^e^	7.18 ± 0.31 ^c^
2	7.2 ± 0.13 ^a^	7.49 ± 0.15 ^e^	8.87 ± 0.13 ^d^	6.44 ± 0.11 ^f^	4.70 ± 0.14 ^h^
3	7.2 ± 0.13 ^a^	7.70 ± 0.17 ^d^	8.73 ± 0.20 ^d^	6.21 ± 0.30 ^h^	5.89 ± 0.16 ^g^
4	7.2 ± 0.13 ^a^	8.12 ± 0.12 ^c^	9.35 ± 0.15 ^b^	7.87 ± 0.12 ^a^	6.57 ± 0.12 ^e^
5	7.2 ± 0.13 ^a^	8.25 ± 0.13 ^c^	9.78 ± 0.16 ^a^	7.21 ± 0.45 ^d^	6.10 ± 0.25 ^f^
6	7.2 ± 0.13 ^a^	7.59 ± 0.23 ^e^	8.60 ± 0.10 ^de^	6.47 ± 0.24 ^f^	6.94 ± 0.20 ^d^
7	7.2 ± 0.13 ^a^	8.52 ± 0.11 ^b^	9.74 ± 0.19 ^a^	7.50 ± 0.28 ^c^	7.54 ± 0.33 ^a^
8	7.2 ± 0.13 ^a^	8.96 ± 0.08 ^a^	9.11 ± 0.14 ^c^	7.70 ± 0.14 ^b^	7.25 ± 0.21 ^b^
9	7.2 ± 0.13 ^a^	8.18 ± 0.16 ^c^	9.20 ± 0.12 ^c^	6.23 ± 0.11 ^g^	6.13 ± 0.18 ^f^
**Spore Cells**			**Dry Films (log_10_ CFU/g)**		
				**4 °C**	**25 °C**
**Sample Code** **	**BI** * (log_10_ CFU/mL)	**AI** * (log_10_ CFU/mL)	**0 Day**	**14 Day**	
1	6.9 ± 0.11 ^a^	6.07 ± 0.12 ^a^	7.44 ± 0.10 ^a^	7.69 ± 0.16 ^ab^	7.79 ± 0.15 ^ab^
2	6.9 ± 0.11 ^a^	5.76 ± 0.14 ^b^	6.04 ± 0.23 ^c^	7.02 ± 0.33 ^cd^	6.64 ± 0.21 ^c^
3	6.9 ± 0.11 ^a^	3.37 ± 0.11 ^h^	4.92 ± 0.16 ^e^	6.34 ± 0.21 ^e^	5.53 ± 0.10 ^e^
4	6.9 ± 0.11 ^a^	2.85 ± 0.08 ^ı^	4.10 ± 0.17 ^g^	6.55 ± 0.23 ^de^	6.76 ± 0.20 ^c^
5	6.9 ± 0.11 ^a^	4.63 ± 0.09 ^d^	5.98 ± 0.14 ^cd^	7.51 ± 0.16 ^bc^	6.82 ± 0.21 ^c^
6	6.9 ± 0.11 ^a^	5.54 ± 0.15 ^c^	6.43 ± 0.25 ^b^	8.20 ± 0.15 ^a^	8.28 ± 0.11 ^a^
7	6.9 ± 0.11 ^a^	4.41 ± 0.13 ^de^	5.87 ± 0.28 ^cd^	6.66 ± 0.17 ^d^	7.36 ± 0.17 ^b^
8	6.9 ± 0.11 ^a^	4.12 ± 0.14 ^f^	5.51 ± 0.18 ^d^	6.10 ± 0.35 ^f^	6.05 ± 0.21 ^d^
9	6.9 ± 0.11 ^a^	3.87 ± 0.09 ^g^	4.56 ± 0.07 ^f^	6.51 ± 0.22 ^de^	7.23 ± 0.57 ^bc^

* BI: Before incubation, the concentration of vegetative cells of *B. clasuii* in the film solutions AI: After 24 h incubation, the concentration of vegetative cells of *B. clasuii* in the film solutions. The superscripts of different letters in a column indicate significant differences among the film samples (Duncan test, *p* < 0.05). ** 1: pH = 8.5, Incubation temperature (IT) = 33, WPC = 6; 2: pH = 8.5, IT = 35, WPC = 8; 3: pH = 8.5, IT = 37, WPC = 10; 4: pH = 9, IT = 33, WPC = 8; 5: pH = 9, IT = 35, WPC = 10; 6: pH = 9.5, IT = 37, WPC = 6; 7: pH = 9.5, IT = 33, WPC = 10; 8: pH = 9.5, IT = 35, WPC = 6; 9: pH = 9.5, IT = 37, WPC = 8.

**Table 3:** The survival of vegetative and spore cells of *B. clausii* in the WPC films after 4 days of storage.

Sample Code *	Survival of Vegetative Cells (%)		Survival of Spore Cells (%)	
	4 °C	25 °C	4 °C	25 °C
1	86.43 ± 2.3 ^a^	87.78 ± 3.2 ^a^	99.36 ± 0.9 ^a^	100.0 ± 0.0 ^a^
2	72.60 ± 3.5 ^d^	52.99 ± 5.6^f^	96.23 ± 0.2 ^c^	100.0 ± 0.0 ^a^
3	71.13 ± 8.2 ^de^	67.47 ± 2.1 ^cd^	98.86 ± 0.6 ^b^	100.0 ± 0.0 ^a^
4	84.17 ± 1.7 ^b^	70.27 ± 4.0 ^c^	99.76 ± 0.5 ^a^	100.0 ± 0.0 ^a^
5	73.72 ± 2.1 ^d^	62.37 ± 2.9 ^e^	95.59 ± 0.8 ^c^	100.0 ± 0.0 ^a^
6	75.23 ± 2.0 ^cd^	80.70 ± 1.8 ^b^	100.0 ± 0.0 ^a^	100.0 ± 0.0 ^a^
7	77.00 ± 5.2 ^c^	77.41 ± 2.7 ^bc^	100.0 ± 0.0 ^a^	100.0 ± 0.0 ^a^
8	84.52 ± 2.4 ^b^	79.58 ± 3.2 ^b^	98.26 ± 0.2 ^b^	99.35 ± 1.0 ^a^
9	67.72 ± 1.9 ^e^	66.63 ± 2.6 ^d^	100.0 ± 0.0 ^a^	99.55 ± 1.2 ^a^

Significant considered at *p* ≤ 0.05, variables at same letters on column indicates non-significant differences, N = 3. * 1: pH = 8.5, IT = 33, WPC = 6; 2: pH = 8.5, IT = 35, WPC = 8; 3: pH = 8.5, IT = 37, WPC = 10; 4: pH = 9, IT = 33, WPC = 8; 5: pH = 9, IT = 35, WPC = 10; 6: pH = 9.5, IT = 37, WPC = 6; 7: pH = 9.5, IT = 33, WPC = 10; 8: pH = 9.5, IT = 35, WPC = 6; 9: pH = 9.5, IT = 37, WPC = 8.

**Table 4:** Properties of WPC-based edible film containing *B. clausii*.

Sample Code *	Thickness (mm)	Tensile Strength (MPa)	Elongation at Break (%)	Water Vapour Permeability (WVP) (g∙mm∙m^−2^∙d^−1^kPa^−1^)	Water Solubility (%)
1	0.22 ± 0.01 ^a^	1.25 ± 0.17 ^c^	10.87 ± 0.88 ^c^	61.44 ± 2.95 ^d^	77.76 ± 1.93 ^ab^
2	0.20 ± 0.01 ^a^	2.63 ± 0.10 ^ab^	18.65 ± 1.20 ^bc^	71.45 ± 3.92 ^bc^	60.27 ± 30.08 ^ab^
3	0.22 ± 0.01 ^a^	1.51 ± 0.06 ^c^	21.06 ± 4.52 ^ab^	95.80 ± 5.08 ^a^	51.47 ± 12.22 ^abc^
4	0.19 ± 0.04 ^a^	2.85 ± 0.91 ^ab^	20.08 ± 2.33 ^ab^	74.96 ± 7.43 ^bc^	59.31 ± 26.53 ^ab^
5	0.22 ± 0.02 ^a^	2.08 ± 0.17 ^bc^	27.63 ± 5.40 ^a^	77.12 ± 7.49 ^b^	53.80 ± 6.82 ^ab^
6	0.18 ± 0.03 ^a^	3.01 ± 0.39 ^ab^	14.94 ± 5.16 ^bc^	45.89 ± 3.52 ^e^	80.02 ± 5.63 ^a^
7	0.21 ± 0.01 ^a^	2.97 ± 0.30 ^ab^	23.05 ± 3.61 ^ab^	60.16 ± 5.59 ^d^	24.49 ± 6.98 ^c^
8	0.19 ± 0.04 ^a^	2.67 ± 0.09 ^ab^	10.32 ± 0.08 ^c^	77.85 ± 7.51 ^b^	24.67 ± 6.21 ^c^
9	0.18 ± 0.02 ^a^	3.14 ± 0.37 ^a^	20.66 ± 3.71 ^ab^	65.22 ± 2.06 ^cd^	50.27 ± 12.35 ^bc^

The superscripts of different letters in a column indicate significant differences among the film samples (Duncan test, *p* < 0.05). * 1: pH = 8.5, IT = 33, WPC = 6; 2: pH = 8.5, IT = 35, WPC = 8; 3: pH = 8.5, IT = 37, WPC = 10; 4: pH = 9, IT = 33, WPC = 8; 5: pH = 9, IT = 35, WPC = 10; 6: pH = 9.5, IT = 37, WPC = 6; 7: pH = 9.5, IT = 33, WPC = 10; 8: pH = 9.5, IT = 35, WPC = 6; 9: pH = 9.5, IT = 37, WPC = 8.

**Table 5:** Inhibition zones of the WPC film solutions containing *B. clausii*.

	Inhibition Zone (mm)			
		Film	Solutions	
Sample Code *	*A. niger*	*P. expansum*	*E. coli*	*S. aureus*
1	11.38 ± 0.48 ^bc^	13.88 ± 2.14 ^b^	9.75 ± 0.29 ^c^	8.88 ± 0.25 ^c^
2	13.63 ± 1.93 ^a^	16.63 ± 1.03 ^a^	11.00 ± 0.71 ^b^	10.00 ± 0.41 ^b^
3	9.88 ± 1.25 ^cd^	12.50 ± 0.82 ^bc^	7.38 ± 0.48 ^d^	9.00 ± 0.71 ^c^
4	11.00 ± 1.87 ^bc^	12.00 ± 1.29 ^c^	7.88 ± 0.25 ^d^	11.00 ± 0.91 ^ab^
5	12.38 ± 0.85 ^ab^	16.88 ± 1.25 ^a^	10.50 ± 0.41 ^bc^	10.13 ± 0.25 ^b^
6	9.88 ± 0.75 ^cd^	10.88 ± 0.95 ^cd^	10.00 ± 0.41 ^c^	11.38 ± 0.85 ^a^
7	8.13 ± 0.48 ^de^	10.13 ± 0.75 ^de^	17.25 ± 0.87 ^a^	11.25 ± 0.65 ^a^
8	6.88 ± 0.48 ^e^	8.63 ± 0.75 ^e^	7.38 ± 0.48 ^d^	10.38 ± 0.75 ^ab^
9	10.50 ± 2.16 ^bc^	8.50 ± 0.00 ^e^	9.75 ± 0.50 ^c^	11.00 ± 0.58 ^ab^

The superscripts of different letters in a column indicate significant differences among the film samples (Duncan test, *p* < 0.05). Control: WPC based film without any bacterial fermentation. * 1: pH = 8.5, IT = 33, WPC = 6; 2: pH = 8.5, IT = 35, WPC = 8; 3: pH = 8.5, IT = 37, WPC = 10; 4: pH = 9, IT = 33, WPC = 8; 5: pH = 9, IT = 35, WPC = 10; 6: pH = 9.5, IT = 37, WPC = 6; 7: pH = 9.5, IT = 33, WPC = 10; 8: pH = 9.5, IT = 35, WPC = 6; 9: pH = 9.5, IT = 37, WPC = 8.

## Data Availability

Data are contained within the article or Supplementary Materials.

## Supplementary Materials

The following supporting information can be downloaded at: https://www.mdpi.com/article/10.3390/polym16233375/s1, Figure S1: Signal/noise ratio results of the number of *B. clausii* vegetative cells in the dry film after 14 days of storage at 4 (a) and 25 °C (b), the number of *B. clausii* spore cells in the dry film after 14 days of storage at 4 (c) and 25 °C (d); Table S1: Analysis of variance on the effect of different parameters and their interactions on the properties of the film (*p*-values for independent variables and interactions).
