# Supplementary material for: A Taguchi Approach for Optimization of Antimicrobial Effect of Whey Protein Based Edible Film Fermented by Bacillus clausii
Source: Polymers (Basel). 2024 Nov 29;16(23):3375. doi: 10.3390/polym16233375 (PMC11644552; doi:10.3390/polym16233375)
Supplement: Supplementary file 1 [file polymers-16-03375-s001.zip › polymers-3291776-supplementary.pdf]

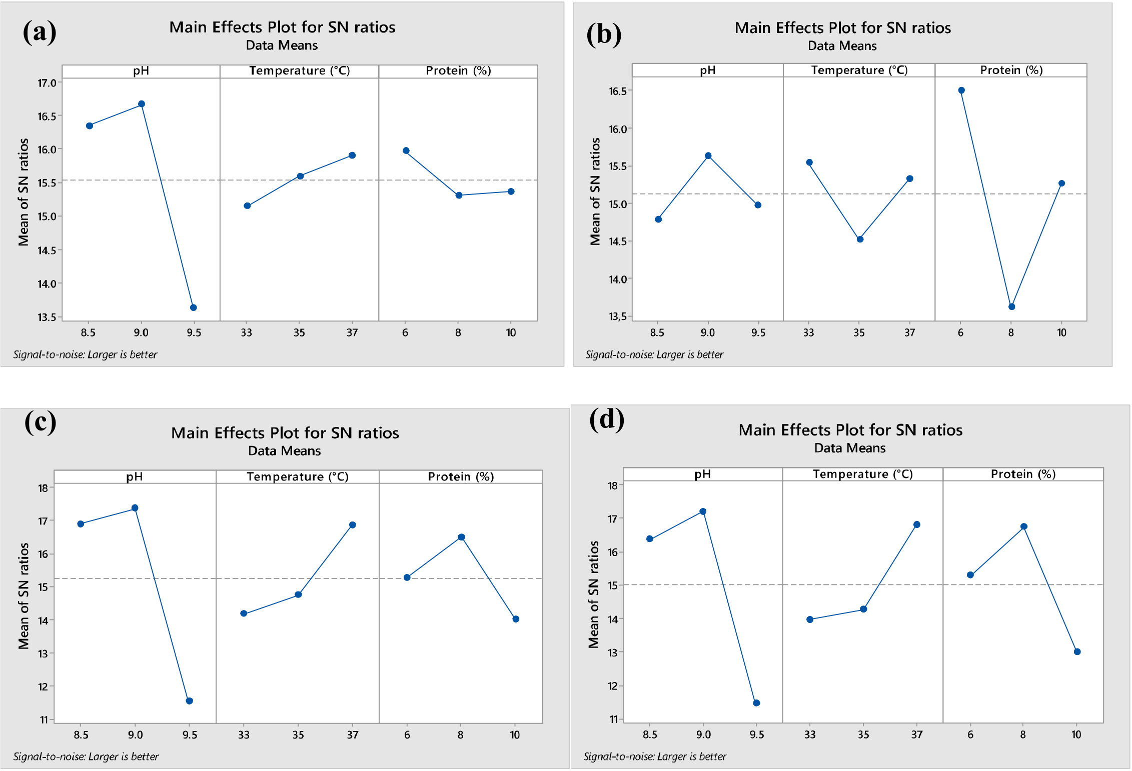

Supplementary Figure S1. Signal/noise ratio results of the number of *B. clausii* vegetative cells in the dry film after 14 days of storage at 4 (a) and 25ºC (b), the number of *B. clausii* spore cells in the dry film after 14 days of storage at 4 (c) and 25ºC (d).

Supplementary Table S1. Analysis of variance on the effect of different parameters and their interactions on the properties of the film (p-values for independent variables and interactions).

Dependent Variable: WVP

Source	Type III Sum of Squares	df	Mean Square	F	P-Sig.	Partial Eta Squared
Corrected Model	2150.231[a]	6	358.372	2.299	0.075	0.657
Intercept	132251.005	1	132251.005	848.449	0.000	1.000
pH	539.856	2	269.928	9.150	0.002	0.949
temperature	459.774	2	229.887	7.792	0.004	0.910
protein	1150.601	2	575.301	19.501	0.000	1.000
pH * temperature	3737.051	4	934.263	31.668	0.000	1.000
pH * protein	3046.224	4	761.556	25.814	0.000	1.000
temperature * protein	3126.306	4	781.577	26.493	0.000	1.000
pH * protein * temperature	2586.450	2	1293.225	43.836	0.000	1.000
Error	3117.476	20	155.874			
Total	137518.711	27				
Corrected Total	5267.707	26				

Dependent Variable: Water solubility

Source	Type III Sum of Squares	df	Mean Square	F	P-Sig.	Partial Eta Squared
Corrected Model	9177.425[a]	8	1147.178	4.964	0.002	0.688
Intercept	77461.686	1	77461.686	335.167	0.000	0.949
pH	5635.974	2	2817.987	12.193	0.000	0.575
temp	927.101	2	463.551	2.006	0.164	0.182
protein	1513.946	2	756.973	3.275	0.061	0.267
pH * temperature	2614.349	4	653.587	2.828	0.056	0.386
pH * protein	2027.505	4	506.876	2.193	0.111	0.328
temperature * protein	6736.377	4	1684.094	7.287	0.001	0.618
pH * protein * temperature	1100.403	2	550.202	2.381	0.121	0.209
Error	4160.050	18	231.114			
Total	90799.161	27				
Corrected Total	13337.475	26				

Dependent Variable: Tensile strength

Source	Type III Sum of Squares	df	Mean Square	F	P-Sig.	Partial Eta Squared
Corrected Model	7.518[a]	8	0.940	6.574	0.005	0.854
Intercept	108.437	1	108.437	758.539	0.000	0.988
pH	4.152	2	2.076	14.521	0.002	0.763
temperature	0.114	2	0.057	0.399	0.682	0.082
protein	1.611	2	0.806	5.635	0.026	0.556
pH * temperature	3.252	4	0.813	5.687	0.015	0.717
pH * protein	1.755	4	0.439	3.069	0.075	0.577
temperature * protein	5.793	4	1.448	10.130	0.002	0.818
pH * temperature * protein	1.641	2	0.820	5.739	0.025	0.560
Error	1.287	9	0.143			
Total	117.242	18				
Corrected Total	8.804	17				

Dependent Variable: % Elongation

Source	Type III Sum of Squares	df	Mean Square	F	P-Sig.	Partial Eta Squared
Corrected Model	511.158[a]	8	63.895	5.199	0.012	0.822
Intercept	6215.753	1	6215.753	505.750	0.000	0.983
pH	51.532	2	25.766	2.096	0.179	0.318
temperature	3.075	2	1.538	0.125	0.884	0.027
protein	435.916	2	217.958	17.734	0.001	0.798
pH * temperature	456.552	4	114.138	9.287	0.003	0.805
pH * protein	23.711	4	5.928	0.482	0.749	0.177
temperature * protein	72.167	4	18.042	1.468	0.290	0.395
pH * temperature * protein	20.635	2	10.318	0.840	0.463	0.157
Error	110.612	9	12.290			
Total	6837.523	18				
Corrected Total	621.770	17				

a. R Squared =0.822 (Adjusted R Squared =0.664)

b. Computed using alpha =0.05
